# STEAP2 Is a Candidate Marker of a 7q/CNA-Associated Iron-Handling Malignant Epithelial State in Lung Adenocarcinoma

**DOI:** 10.3390/cancers18183050

**Published:** 2026-09-20

**Authors:** Xiang Gao, Qiang Liu, Lei Xiong, Jing Luo, Yi Shen

**Affiliations:** Department of Cardiothoracic Surgery, Jinling Hospital, Affiliated Hospital of Medical School, Nanjing University, Nanjing 210002, China; dr_gaoxiang@163.com (X.G.); liuuqiang1994@163.com (Q.L.); threestone1983@163.com (L.X.)

**Keywords:** lung adenocarcinoma, non-small cell lung cancer, STEAP2, single-cell RNA sequencing, copy-number alteration, iron handling, epithelial plasticity, Squamoid subtype

## Abstract

**Background/Objectives:** Single-gene markers may help translate complex malignant epithelial states into bulk and tissue studies, but they must be distinguished from copy-number surrogates and prognostic biomarkers. We evaluated STEAP2 as a candidate marker of a lung adenocarcinoma (LUAD) malignant epithelial state. **Methods:** We integrated TCGA-LUAD bulk expression, subtype, copy-number and survival data with GSE131907 author-annotated malignant cells and GSE148071 CNV-high epithelial-like cells. Analyses included sample-level paired comparisons, sequencing-depth-adjusted mixed-effects models, cutoff and continuous-expression sensitivity analyses, and a pre-specified 7q gene benchmark. **Results:** STEAP2-high TCGA tumors were enriched for the Squamoid expression subtype (59.6% versus 23.3%; odds ratio, 4.69; 95% confidence interval [CI], 2.06–10.69). Iron-handling was the most reproducible single-cell feature: paired STEAP2-positive minus STEAP2-negative differences were 0.779 (95% CI, 0.605–0.952) in GSE131907 and 0.340 (95% CI, 0.304–0.377) in GSE148071, with consistent direction after removing STEAP2 from the score. Metabolic, interferon/major histocompatibility complex and EMT/YAP-like programs were context-dependent. STEAP2 ranked in the top decile of 106 evaluable 7q-window genes (rank fraction, 0.099) and retained state-composite association after adjustment for a 7q copy-number proxy and canonical Squamoid markers. The survival association attenuated after stage adjustment (hazard ratio, 1.17; *p* = 0.359). **Conclusions:** STEAP2 is a candidate marker of a 7q/copy-number-associated iron-handling malignant epithelial state. The evidence supports state identification rather than prognosis, therapeutic targeting or causal driver claims.

## Simple Summary

Single-cell studies identify complex tumor-cell states that are difficult to transfer to routine tissue studies. We asked whether STEAP2, a chromosome 7q metal-reductase gene, could serve as a simple indicator of a reproducible malignant epithelial state in lung adenocarcinoma. Across one bulk-tumor cohort and two single-cell cohorts, iron-handling was the most stable feature associated with STEAP2 at the sample level. STEAP2 ranked within the top decile of evaluable genes in its regional 7q benchmark, although its association with the Squamoid expression subtype partly reflected the broader copy-number background. STEAP2 was not independently associated with survival after adjustment for tumor stage. The findings therefore support STEAP2 as a candidate state-identification marker rather than a proven driver, prognostic biomarker or treatment target. Tissue-level, spatial and functional studies are required before clinical use.

## 1. Introduction

Lung adenocarcinoma (LUAD), a major histological subtype of non-small cell lung cancer (NSCLC), comprises genetically and phenotypically heterogeneous tumors [1,2]. Beyond differences between patients, malignant epithelial cells within the same tumor can occupy distinct proliferative, metabolic, inflammatory and plasticity-associated states [3,4]. These states may influence tumor evolution, treatment adaptation and interactions with the tumor microenvironment, yet they can be obscured when expression is averaged across bulk tissue. Single-cell RNA sequencing (scRNA-seq) has improved the resolution of this heterogeneity and revealed recurrent malignant-cell programs across primary, metastatic and treatment-exposed NSCLC samples [5,6,7].

Most malignant epithelial states are defined by unsupervised clusters, inferred copy-number profiles or multi-gene expression signatures [4,5,6]. These approaches capture complex biology, but their output depends on analytical choices and is not readily transferred to routine tissue assays. A single-gene marker that reproducibly identifies a biologically coherent malignant-cell state could provide a simpler bridge between scRNA-seq, bulk cohorts and protein-based tissue analysis. Such a marker must nevertheless be distinguished from a causal driver: its expression may reflect chromosomal context, cell-state regulation or both. Establishing its value therefore requires cross-cohort validation, copy-number-aware analysis and explicit testing of whether the expression signal contains information beyond regional genomic alteration.

STEAP2 encodes a member of the six-transmembrane epithelial antigen of the prostate family. Functional studies of this family identified STEAP2 as a ferrireductase and cupric reductase capable of promoting cellular iron and copper uptake in vitro [8]. STEAP2 was originally mapped to chromosome 7q21 and characterized as a membrane-associated protein with prominent expression in the prostate epithelium [9]. Subsequent work has investigated STEAP2 most extensively in prostate cancer, where altered expression has been linked to invasive phenotypes [10].

Its relationship to intratumoral malignant epithelial states in LUAD remains incompletely defined. This question is particularly relevant because recurrent copy-number alterations can influence regional gene dosage and transcription in lung cancer [2,11], while dosage compensation and other regulatory processes can prevent expression from being reducible to copy number alone [12]. It is, therefore, unclear whether STEAP2 expression in LUAD merely reports 7q dosage or identifies a more specific transcriptional state involving metabolic stress, iron handling, epithelial plasticity and immune-interaction programs.

Here, we integrated TCGA-LUAD bulk RNA-seq and copy-number data with two independent NSCLC scRNA-seq cohorts to define the transcriptional context of STEAP2 at tumor and malignant-cell resolution. GSE131907, which includes author-annotated malignant cells and broad cell-type annotations, was used for discovery [5]; GSE148071 was used for independent validation within a conservatively defined epithelial-like compartment with high inferred copy-number variation (CNV-high) [6]. We combined signature scoring, differential-expression analysis, pre-ranked gene-set enrichment analysis, copy-number-aware sensitivity models and focused regulon analysis to distinguish shared from cohort-dependent features. Transcript-based ligand-receptor potential and public immunohistochemistry data were examined only as supplementary exploratory layers. This framework was designed to test whether STEAP2 serves as a 7q copy-number-alteration-associated but non-redundant marker of a recurrent malignant epithelial state, while keeping causal and clinical claims within the limits of observational public-cohort data.

## 2. Materials and Methods

### 2.1. Study Design and Datasets

This study integrated public bulk RNA-seq, copy-number, survival, single-cell RNA-seq (scRNA-seq) and immunohistochemistry resources to evaluate STEAP2 as a candidate marker of a malignant epithelial state in LUAD/NSCLC. TCGA-LUAD was used for bulk expression, subtype, pathway, copy-number and survival analyses. GSE131907 was used as the primary scRNA-seq discovery cohort because it provides author-annotated malignant cells and broad cell-type annotations. GSE148071 was used for independent validation after marker-based epithelial-like cell inference and lightweight inferCNV filtering. Human Protein Atlas (HPA) pathology data assessed STEAP2 immunohistochemistry feasibility. Cohort roles, target-cell definitions and limitations are summarized in Table 1 and Table S2.

All analyses used de-identified public datasets. No new human participants were recruited, and no protected health information was analyzed.

Because this was a retrospective secondary analysis of public cohorts, study size was determined by the availability of samples or cells with the required expression, annotation and copy-number information rather than by an a priori power calculation. All eligible samples or cells meeting each analysis-specific definition were included. No imputation was performed because benchmark performance is method- and dataset-dependent and imputation can alter downstream expression structure [13]. Analyses used complete available data for the variables required in each comparison; genes absent from a given dataset were omitted from the corresponding module score, and analysis-specific denominators are reported in the Results, Table 1 and Table S2–S13.

### 2.2. TCGA-LUAD Bulk Expression and Subtype Analysis

TCGA-LUAD normalized RNA-seq expression and clinical annotation matrices were obtained from the UCSC Xena platform (TCGA-LUAD cohort; accessed on 10 July 2026) [14]. Primary tumor samples with available STEAP2 expression were retained for bulk analyses. Tumor and normal samples were identified from TCGA barcode suffixes, and TCGA-LUAD tumors were used for expression-subtype and pathway-score analyses.

Tumors were stratified by STEAP2 expression quartiles. The lowest quartile was defined as STEAP2-low Q1, the upper quartile as STEAP2-high Q4, and the middle quartiles as Q2–Q3. Squamoid enrichment was evaluated by comparing Q4 with Q1 tumors in a 2 × 2 contingency table, with odds ratios and 95% confidence intervals calculated using Haldane–Anscombe correction.

TCGA module scores were calculated as the mean z-scored expression of available genes in each module. Modules included metabolic/redox, glycolysis/lactate, EMT/YAP-like, partial EMT, CAF/ECM, proliferation, immune, IFN-gamma, antigen-presentation, checkpoint and myeloid programs. ICI-related scores were treated as transcriptome-based proxies, not formal TIDE or IPS outputs.

### 2.3. TCGA Copy-Number Analysis

TCGA-LUAD GISTIC2 gene-level thresholded and continuous copy-number matrices were obtained from UCSC Xena and merged with TCGA-LUAD expression scores [14,15]. Copy-number analysis included 512 tumors with matched STEAP2 expression and copy-number data. STEAP2 copy-number gain was defined from gene-level calls, and continuous STEAP2 CNA values were used for correlation and regression analyses.

A local 7q expression proxy was calculated from genes in a pre-defined 7q window around STEAP2. Spearman correlation assessed associations among STEAP2 CNA, STEAP2 expression and the 7q proxy. To benchmark STEAP2 against regional passenger genes, all annotated genes within the STEAP2-centered 7q window were ranked by their association with a state-composite score in TCGA-LUAD. Genes absent from the expression matrix were excluded. The pre-specified interpretation rule classified STEAP2 as a top-decile candidate marker if it ranked within the top 10% of evaluable 7q genes, a representative surrogate if it ranked within the top 25%, and a redundant regional marker if it ranked below the top 25%.

Multivariable linear models tested whether STEAP2 expression retained associations with transcriptional program scores after accounting for STEAP2 CNA or the 7q proxy, with additional sensitivity models including expression subtype, canonical Squamoid marker score and transcriptome-based stromal/immune surrogates. Logistic regression modeled Squamoid versus non-Squamoid subtype as a function of standardized STEAP2 expression, canonical Squamoid marker score and STEAP2 CNA or the 7q proxy.

### 2.4. TCGA Survival Sensitivity Analysis

Overall-survival analyses were performed in TCGA-LUAD as a claim-calibration step rather than as a primary prognostic-biomarker analysis. Cox proportional-hazards models tested continuous standardized STEAP2 expression, STEAP2 Q4 versus all other tumors, and STEAP2 Q4 versus Q1. Models were run unadjusted and then adjusted for pathological stage, with additional sensitivity adjustment for age, sex and available ABSOLUTE purity estimates where data were available. Survival results were used to determine whether prognostic wording was justified.

### 2.5. GSE131907 Single-Cell Discovery Analysis

For GSE131907, the author-provided annotation file and raw UMI matrix were obtained from the Gene Expression Omnibus (GEO) and used with the source-study annotations [5,16,17]. Cells annotated as malignant cells were retained as the discovery population, yielding 24,784 author-annotated malignant cells.

STEAP2-positive malignant cells were defined by non-zero STEAP2 raw UMI expression after log1p transformation. STEAP2-high malignant cells were defined as cells with STEAP2 log1p raw UMI values at or above the 75th percentile among STEAP2-positive malignant cells. This cutoff was 1.099 on the log1p raw UMI scale. The 75th percentile among STEAP2-positive cells was chosen to define a high-confidence positive subset within the expressing population, avoiding inclusion of borderline low-UMI cells. Cells below this threshold, including STEAP2-negative cells, were treated as STEAP2-low or negative for the stringent high-versus-other comparison.

For signature scoring, raw UMI values were log1p-transformed, genes were z-scored across malignant cells, and each score was calculated as the mean z-scored expression of available signature genes. The metabolic signature used in the main GSE131907 analysis excluded STEAP2 to avoid circular inflation. Signature gene sets are listed in Supplementary Figure S7 and Supplementary Table S1.

Differential expression between STEAP2-high and other malignant cells was calculated from the raw UMI matrix. For each gene, group means, detection percentages, mean log1p expression and average log1p fold change were calculated. *p* values were estimated using an approximate Welch z-test on log1p raw UMI values and adjusted by the Benjamini–Hochberg method. Upregulated genes required average log1p fold change greater than 0.10, detection difference greater than 2 percentage points and FDR less than 0.05; downregulated genes used corresponding negative thresholds. Outputs were used for visualization, redox/iron watch-gene evaluation and pre-ranked GSEA.

### 2.6. GSE131907 Quality Control and inferCNV Sensitivity Analysis

Quality-control analyses calibrated the GSE131907 discovery contrast. Differential-expression directionality was summarized to avoid overinterpreting the small downregulated gene set. COL1A1 and related ECM/CAF-like markers were examined for possible ambient RNA, stromal contamination or doublet effects, and canonical YAP/TEAD target genes were evaluated to calibrate the EMT/YAP-like interpretation.

A lightweight inferCNV-like sensitivity analysis tested whether STEAP2-associated programs persisted in CNV-malignant-like cells [5,6]. The analysis included all author-annotated malignant cells and 10,000 randomly sampled non-epithelial reference cells. Genes were ordered by genomic position, and cell-level CNV burden was estimated from smoothed expression residuals relative to the reference. A CNV-malignant-like threshold was defined from the reference-cell burden distribution; 14,696 malignant cells met this threshold. Signature effect sizes were recalculated in all malignant cells, CNV-malignant-like malignant cells and CNV-low malignant cells.

### 2.7. GSE148071 Epithelial-like Validation Analysis

GSE148071 data were obtained from GEO and analyzed with the source-study context [6,16,17]. Because comparable author-provided malignant-cell annotations were unavailable, epithelial-like cells were inferred using epithelial, immune and stromal marker scores, yielding a marker-inferred epithelial-like target population of 57,546 cells.

Lightweight inferCNV filtering was then applied to identify CNV-high epithelial-like cells. Cell-level CNV burden was estimated from gene-ordered expression residuals relative to reference-like cells. The final validation compartment included 6912 CNV-high epithelial-like cells, comprising 820 STEAP2-positive and 6092 STEAP2-negative cells.

For differential expression, raw counts were normalized to counts per 10,000 UMIs and log1p-transformed. Mean log1p CP10K expression, detection percentage, Welch t statistic, Cohen’s d and Benjamini–Hochberg FDR were calculated between STEAP2-positive and STEAP2-negative CNV-high epithelial-like cells. Signature scores were calculated as the mean of within-dataset z-scored expression values across available genes. Full outputs are provided in Supplementary Table S6.

### 2.8. Sample-Level, Cutoff and Sequencing-Depth Robustness Analyses

Sample-level robustness analyses were performed in both GSE131907 and GSE148071 to reduce cell-level pseudoreplication. For each cohort, within-sample paired analyses required at least 10 STEAP2-positive and 10 STEAP2-negative cells in the relevant compartment. For each informative sample and signature, the mean STEAP2-positive minus STEAP2-negative difference was calculated and summarized with 95% confidence intervals. The pre-specified cross-cohort interpretation rule defined a signal as consistent if both cohorts had the same direction and at least one 95% CI did not cross zero, weak if both cohorts had the same direction but both confidence intervals crossed zero, and absent if the directions differed.

To harmonize the primary cross-cohort comparison, sample-level analyses used STEAP2-positive versus STEAP2-negative definitions in both datasets. Cutoff sensitivity analyses additionally evaluated the 50th, 75th and 90th percentiles of STEAP2 expression within the expressing population. Because many GSE148071-positive cells contained one STEAP2 UMI, its 50th- and 75th-percentile thresholds were identical. Continuous-expression sensitivity analyses tested associations between STEAP2 expression and signature scores without dichotomizing cells. Sequencing-depth sensitivity was assessed using mixed-effects models adjusted for total UMI counts and detected genes, with the sample modeled as a grouping factor where supported by the available metadata. Logistic models also tested whether STEAP2 detection was associated with these depth metrics.

For the matched-expression detection benchmark, gene-level UMI abundance and non-zero detection fractions were calculated across the target cells in each cohort. STEAP2 was compared with the 100 nearest genes under two matching metrics: mean UMI across all target cells and mean UMI among expressing cells. Mitochondrial and ribosomal genes and genes detected in fewer than 10 cells were excluded. The STEAP2 detection percentile was the proportion of matched genes with a detection fraction less than or equal to that of STEAP2; values from 0.10 to 0.90 were descriptively classified as within the matched-gene distribution. This benchmark tests whether detection is unusually sparse for the observed abundance but cannot distinguish technical dropout from biological on/off expression. Together, these analyses were used to distinguish robust signature associations from low-UMI dropout or depth-related artifacts; the observed detection fraction itself was not assumed to be purely biological.

### 2.9. Pre-Ranked Gene-Set Enrichment Analysis

Pre-ranked GSEA was performed using gseapy, following the GSEA framework and MSigDB resources [18,19,20]. Gene sets were obtained from MSigDB Hallmark 2020, KEGG 2021 Human and Reactome 2022. TCGA-LUAD genes were ranked by Spearman correlation with STEAP2 expression, GSE131907 genes by average log1p fold change, and GSE148071 genes by Welch t statistic.

GSEA used 500 permutations, a minimum gene-set size of 10 and a maximum gene-set size of 500. Results are reported as normalized enrichment scores and FDR q values. Positive enrichment indicates association with STEAP2-high or STEAP2-positive groups. Software-reported FDR q values of zero reflect the numerical resolution of the empirical null estimate rather than a literal zero probability; these values are displayed as q < 0.001 in manuscript figures while the original output is retained in the source tables.

### 2.10. Focused pySCENIC Regulon Analysis

Focused regulon analysis used pySCENIC-derived AUCell matrices generated for selected transcription factors and regulon sets [21,22]. In GSE131907, STEAP2-high malignant cells were compared with STEAP2-low or negative malignant cells. In GSE148071, STEAP2-positive CNV-high epithelial-like cells were compared with STEAP2-negative cells. Differential regulon activity was summarized using mean activity differences, Cohen’s d and Spearman correlation with STEAP2 expression.

Bootstrap subsampling evaluated regulon stability by sampling 80% of the smaller STEAP2 group and recalculating Cohen’s d. A regulon was considered stable if the 95% bootstrap interval did not cross zero and at least 95% of the iterations retained the same direction. This was a focused pySCENIC regulon analysis with bootstrap stability assessment, not a full unbiased SCENIC rerun.

### 2.11. Focused Ligand-Receptor Scoring

Focused ligand-receptor analysis was performed only in GSE131907, which includes author-annotated malignant cells and broad cell-type annotations. Malignant cells were split into STEAP2-high and STEAP2-low or negative senders. Recipient groups included myeloid cells, T/NK cells, B cells, fibroblast/CAF cells, endothelial cells and mast cells.

A curated CellPhoneDB-style ligand-receptor framework was used [23]. Focused axes included HLA/MHC, ICAM/adhesion, MIF, chemokines, TGFB, SPP1, VEGF, EGFR/ERBB, THBS/CD47 and exploratory ECM/integrin pairs. Interaction scores were calculated as mean log1p ligand expression in malignant sender cells multiplied by mean log1p receptor expression in the recipient group, in pooled cells and informative samples. This analysis estimates transcript-based interaction potential and does not establish spatial contact or functional signaling.

### 2.12. HPA Immunohistochemistry Feasibility Analysis

STEAP2 protein-detection feasibility was evaluated using HPA lung cancer pathology data. Lung cancer immunohistochemistry categories were extracted for antibody HPA029115 and summarized as high, medium, low or not detected. These data were used only to assess feasibility of antibody-based STEAP2 detection, not to validate the scRNA-seq-inferred STEAP2-positive malignant-cell state or its spatial organization.

### 2.13. Statistical Analysis and Figure Generation

Continuous variables were compared using effect sizes and analysis-specific tests. Cohen’s d summarized standardized differences between two groups. Because target-cell populations and reference-group compositions differed among datasets, Cohen’s d values were interpreted within each dataset-specific contrast and were not treated as directly interchangeable across cohorts. Spearman correlation was used for rank-based associations. *p* values from differential-expression and model-based analyses were adjusted using the Benjamini–Hochberg method where multiple testing was performed [24]. Bootstrap resampling was used for regulon stability summaries [25]. Contingency analyses or logistic regression were used for subtype enrichment analyses where appropriate. All tests were two-sided unless otherwise stated.

Analyses were performed using R version 4.5.1 and Python version 3.10.12. Primary tabular outputs are provided as supplementary tables and figure source-data files. Figures were generated from analysis-specific TSV outputs using R/ggplot2 or Python/matplotlib with publication-style formatting. Editable SVG, vector PDF, PNG and TIFF versions were exported for main and supplementary figures. Source data for each figure panel are listed in Supplementary Table S13. Signature definitions and gene sets are provided in Supplementary Table S1. Blank cells in supplementary source-data tables indicate variables not applicable to that table section.

## 3. Results

### 3.1. Study Design and Cohort Definitions

We assembled a multi-layer public-cohort framework to evaluate STEAP2 as a candidate marker of a LUAD/NSCLC malignant epithelial state. The framework included TCGA-LUAD bulk RNA-seq, expression subtype, copy-number and survival data; GSE131907 single-cell RNA-seq; and GSE148071 single-cell RNA-seq (Table 1, Tables S1 and S2). GSE131907 served as the primary single-cell discovery cohort because it included author-annotated malignant cells. GSE148071 served as an independent validation cohort in CNV-high epithelial-like cells because comparable author-defined malignant-cell annotations were not available. Human Protein Atlas (HPA) pathology data were used only to assess antibody and protein-detection feasibility.

STEAP2 groups were defined within each data layer. In TCGA-LUAD, tumors were stratified by STEAP2 expression quartiles, with Q4 defined as STEAP2-high and Q1 as STEAP2-low. In GSE131907, analyses used both STEAP2-positive malignant cells and a more stringent STEAP2-high malignant-cell subset. In GSE148071, validation compared STEAP2-positive and STEAP2-negative cells within the CNV-high epithelial-like compartment. This framework separated bulk expression context, single-cell malignant-state discovery, copy-number association, and independent single-cell validation.

### 3.2. TCGA-LUAD Links High STEAP2 Expression to Squamoid Subtype and Stress-Associated Programs

**Figure 1:**
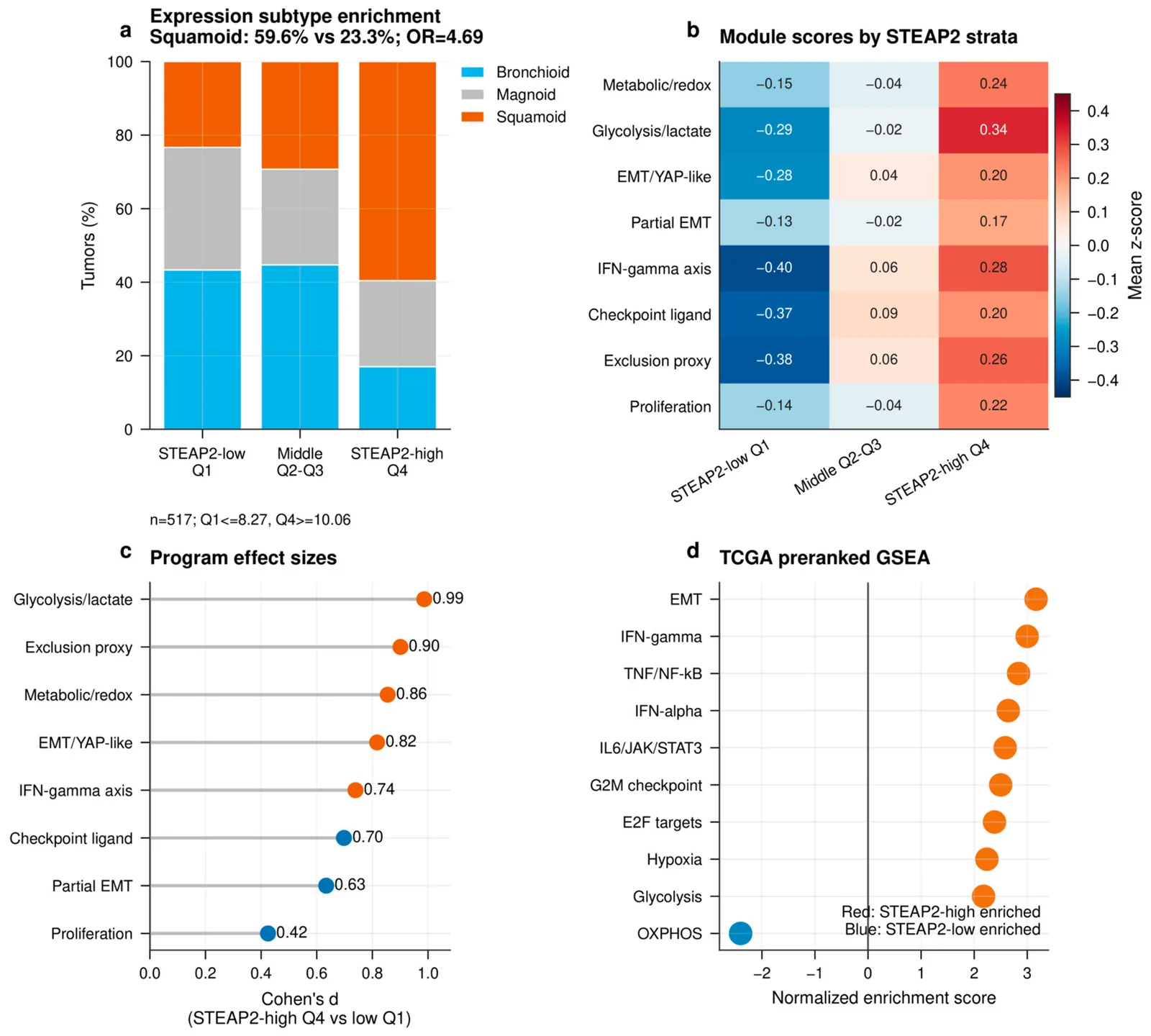
TCGA-LUAD bulk analysis links STEAP2 expression to Squamoid subtype and stress-associated transcriptional programs. (**a**) Distribution of TCGA-LUAD expression subtypes across STEAP2 expression strata. Tumors were stratified by STEAP2 expression quartiles, with Q1 defined as STEAP2-low and Q4 as STEAP2-high. Squamoid enrichment was evaluated by comparing STEAP2-high Q4 with STEAP2-low Q1 tumors. (**b**) Mean module scores across STEAP2 expression strata in TCGA-LUAD. Scores summarize selected metabolic/redox, glycolytic, EMT/YAP-like, inflammatory, antigen-presentation, and immune-related transcriptional programs. (**c**) Effect sizes for selected TCGA-LUAD transcriptional programs comparing STEAP2-high Q4 with STEAP2-low Q1 tumors. Bars indicate Cohen’s d. (**d**) Pre-ranked GSEA of genes ranked by association with STEAP2 expression in TCGA-LUAD. Dot position indicates normalized enrichment score (NES), dot size indicates −log10 FDR, and color indicates enrichment direction. GSEA, gene set enrichment analysis; LUAD, lung adenocarcinoma; NES, normalized enrichment score; FDR, false discovery rate.

We first evaluated STEAP2 in TCGA-LUAD to define its bulk transcriptomic context. STEAP2-high tumors were enriched for the Squamoid expression subtype compared with STEAP2-low tumors (28/47, 59.6% vs. 14/60, 23.3%; odds ratio [OR] = 4.69, 95% confidence interval [CI] = 2.06–10.69; Figure 1a; Supplementary Table S3). This subtype enrichment established Squamoid context as a major bulk-level feature of STEAP2-high LUAD.

STEAP2-high tumors also showed higher module scores for metabolic/redox, glycolysis/lactate, EMT/YAP-like, interferon-associated, antigen-presentation, hypoxia, and inflammatory signaling programs (Figure 1b,c; Supplementary Table S3). Pre-ranked GSEA further identified enrichment of EMT, interferon gamma response, TNF/NF-kB signaling, and glycolysis among genes positively associated with STEAP2 (Figure 1d; Supplementary Table S3). These bulk findings defined the initial transcriptional context, but they did not by themselves establish whether STEAP2 was a cell-state marker, a 7q copy-number surrogate, or a prognostic biomarker.

### 3.3. STEAP2-High Malignant Cells in GSE131907 Show Dominant Metabolic and Redox Features

**Figure 2:**
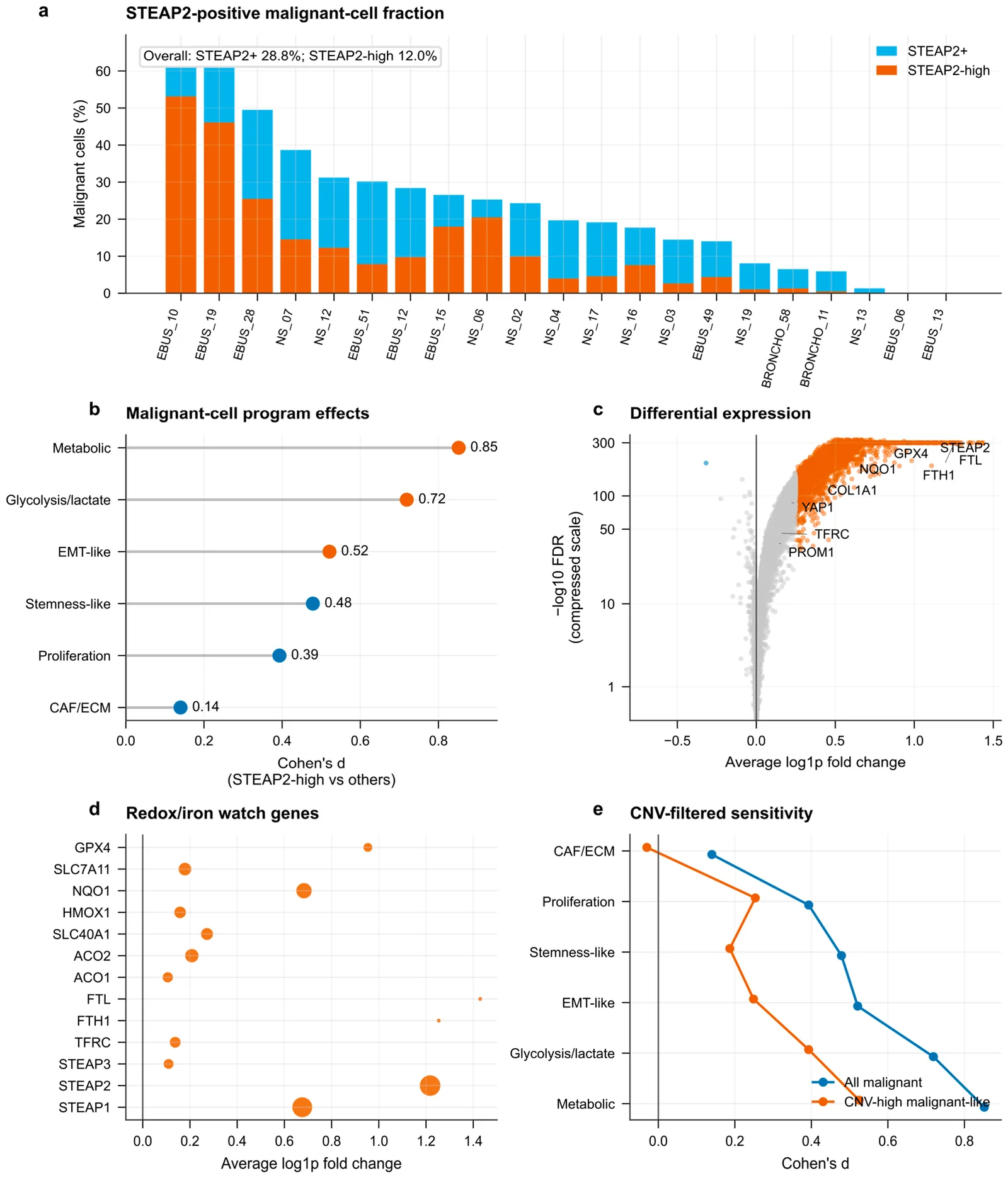
GSE131907 identifies a STEAP2-high malignant-cell state dominated by metabolic/redox and partial EMT-like features. (**a**) Observed non-zero STEAP2 detection and STEAP2-high fractions across GSE131907 samples within author-annotated malignant cells. STEAP2-high cells were defined using a more stringent expression threshold among STEAP2-positive malignant cells. These detection fractions are sequencing-depth dependent and are not estimates of the true biological prevalence of the state. (**b**) Signature effect sizes comparing STEAP2-high malignant cells with other malignant cells in GSE131907. Bars indicate Cohen’s d for metabolic, glycolytic, EMT-like, stemness-like, proliferation, and CAF/ECM-like signatures. (**c**) Differential-expression landscape for STEAP2-high malignant cells versus other malignant cells. The y axis uses a compressed significance scale because many genes had extremely small FDR values. Highlighted genes indicate selected state-associated, metabolic, redox/iron, or epithelial markers. (**d**) Redox and iron-handling watch genes in the STEAP2-high versus other malignant-cell comparison. Values summarize differential-expression statistics for curated redox, ferroptosis-related, and iron-handling genes. (**e**) CNV-filtered sensitivity analysis in GSE131907. Signature effect sizes are shown before and after lightweight inferCNV-based filtering of malignant cells. CNV, copy-number variation; EMT, epithelial–mesenchymal transition; CAF, cancer-associated fibroblast; ECM, extracellular matrix.

We next tested whether the TCGA signal corresponded to a malignant epithelial state at single-cell resolution. In GSE131907, non-zero STEAP2 transcripts were detected in 7130 of 24,784 author-annotated malignant cells (28.8%). A more stringent STEAP2-high subset included 2973 malignant cells (12.0%) (Figure 2a; Supplementary Table S2). Thus, STEAP2 was not uniformly detected across malignant cells; the observed detection fraction is sequencing-depth dependent and should not be interpreted as the true biological prevalence of the state.

Compared with other malignant cells, STEAP2-high malignant cells showed the largest effects for metabolic and glycolytic signatures. Effect sizes were smaller for EMT-like and stemness-like signatures, and weak for CAF/ECM-like scoring (metabolic d = 0.852; glycolysis/lactate d = 0.719; EMT-like d = 0.521; stemness-like d = 0.479; CAF/ECM-like d = 0.140; Figure 2b; Supplementary Table S4). The discovery cohort therefore supported metabolic remodeling as the dominant STEAP2-associated single-cell feature.

Differential-expression analysis of STEAP2-high versus other malignant cells identified broad upregulation of metabolic, stress-response, redox, and epithelial-state-associated genes (Figure 2c,d; Supplementary Table S4). Redox and iron-handling watch genes were also represented among STEAP2-associated transcripts. The differential-expression profile was strongly asymmetric, with thousands of upregulated genes and only a small downregulated set. We therefore did not infer a coordinated suppressed pathway from the downregulated genes (Supplementary Figure S1; Supplementary Table S5).

Several quality-control analyses refined this malignant-cell interpretation. COL1A1 and related ECM signals were present in the differential-expression output, but the CAF/ECM module had a weak malignant-cell effect size. This reduced support for a strong tumor-intrinsic CAF/ECM conclusion (Figure 2b and Figure S1; Supplementary Table S5). YAP1 and YAP/TEAD-adjacent markers contributed to an EMT/YAP-like score, but canonical downstream evidence was incomplete. We therefore refer to this program as EMT/YAP-like rather than definitive YAP/TEAD activation.

### 3.4. Sample-Level and Depth-Adjusted Analyses Identify Iron-Handling as the Most Reproducible Single-Cell Feature

Because cell-level contrasts can inflate apparent precision when many cells come from the same patient, we reanalyzed key single-cell signatures at the sample level. We pre-specified paired analyses requiring at least 10 STEAP2-positive and 10 STEAP2-negative cells per sample and classified cross-cohort reproducibility as consistent when both cohorts had the same direction and at least one 95% CI did not cross zero.

**Figure 3:**
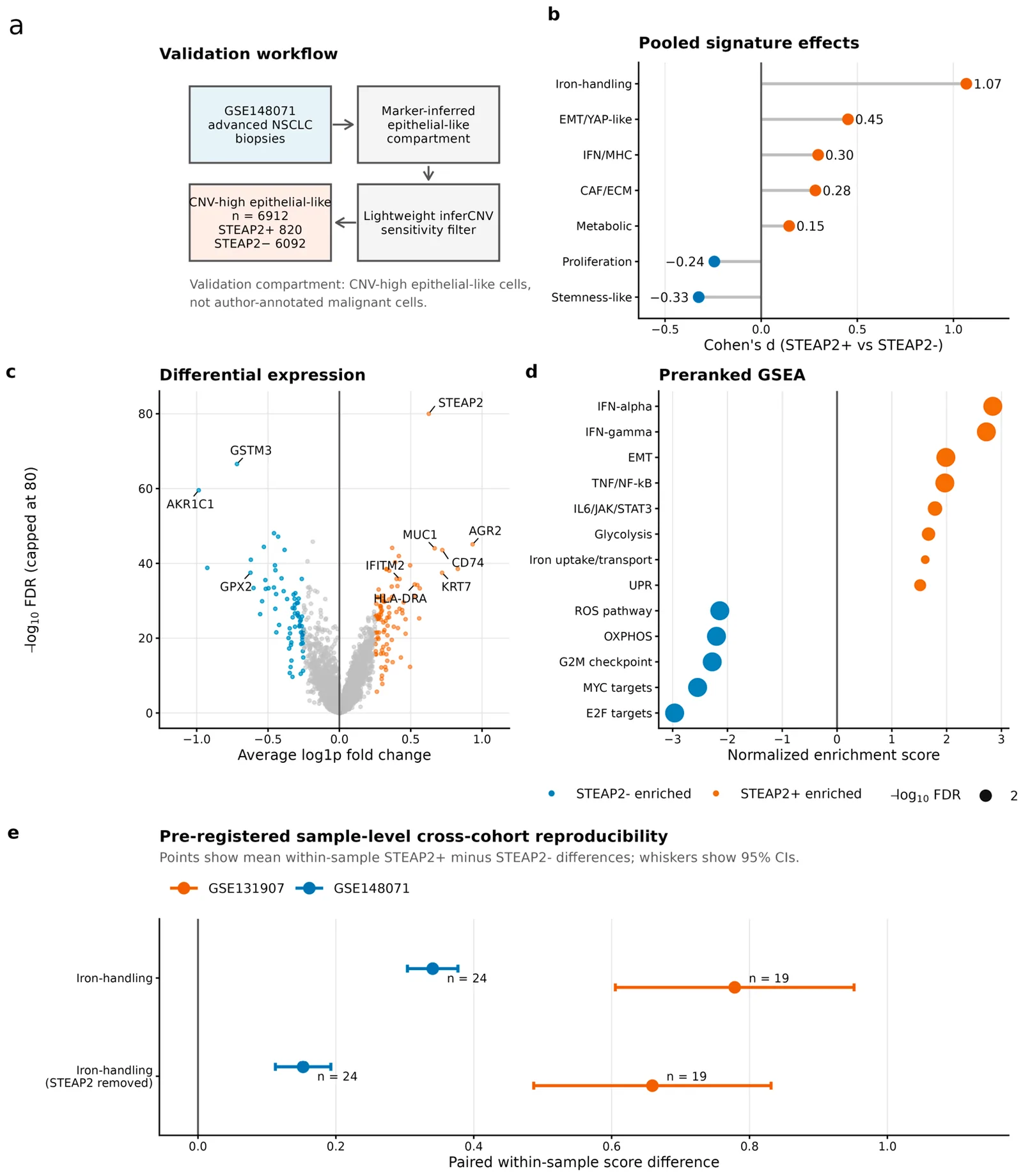
GSE148071 supports the STEAP2-associated state in CNV-high epithelial-like cells. (**a**) Validation workflow for GSE148071. Marker-inferred epithelial-like cells were filtered using lightweight inferCNV to define CNV-high epithelial-like cells, followed by comparison of STEAP2-positive and STEAP2-negative cells. (**b**) Signature effect sizes comparing STEAP2-positive and STEAP2-negative CNV-high epithelial-like cells. Bars indicate Cohen’s d for iron-handling, EMT/YAP-like, IFN/MHC, metabolic, proliferation, stemness-like, and related signatures. (**c**) Differential-expression landscape for STEAP2-positive versus STEAP2-negative CNV-high epithelial-like cells. Highlighted genes indicate selected epithelial, inflammatory, iron-handling, and state-associated markers. (**d**) Pre-ranked GSEA of STEAP2-positive versus STEAP2-negative CNV-high epithelial-like cells. Dot position indicates NES, dot size indicates −log10 FDR, and color indicates enrichment direction. (**e**) Cross-cohort sample-level paired estimates for iron-handling and the iron-handling score recalculated without STEAP2. Points show mean within-sample STEAP2-positive minus STEAP2-negative differences; whiskers show 95% confidence intervals. Samples were eligible when both compartments contained at least 10 cells. IFN, interferon; MHC, major histocompatibility complex; NSCLC, non-small cell lung cancer.

Iron-handling passed this pre-specified reproducibility criterion. The paired STEAP2-positive minus STEAP2-negative difference was 0.779 in GSE131907 (95% CI, 0.605–0.952; 19 eligible samples) and 0.340 in GSE148071 (95% CI, 0.304–0.377; 24 eligible samples; Figure 3e; Supplementary Table S7). To exclude circularity from STEAP2 itself, we recalculated the iron-handling signature after removing STEAP2. The direction remained consistent in both cohorts, with paired differences of 0.659 in GSE131907 (95% CI, 0.487–0.831) and 0.153 in GSE148071 (95% CI, 0.112–0.193). Thus, the iron-handling program was not merely a mathematical reflection of including STEAP2 in the score.

Depth-adjusted mixed-effects models further supported the iron-handling association. After adjustment for total UMI counts and detected genes, STEAP2 positivity remained associated with iron-handling scores in both GSE131907 (beta = 0.244, 95% CI, 0.236–0.252) and GSE148071 (beta = 0.298, 95% CI, 0.279–0.317). The iron-handling association remained positive across the positive, 75th-percentile and 90th-percentile definitions and in continuous-expression analyses. Pooled Spearman correlations between continuous STEAP2 expression and iron-handling scores were 0.514 in GSE131907 and 0.299 in GSE148071; the median within-sample correlations were 0.469 and 0.340, respectively. In contrast, EMT/YAP-like effects were not retained after UMI/nGene adjustment in either cohort.

STEAP2 detection itself was associated with the number of detected genes after depth adjustment, indicating that the 28.8% detection fraction cannot be interpreted as purely biological heterogeneity. We therefore benchmarked STEAP2 against the 100 nearest non-mitochondrial, non-ribosomal genes by mean UMI across all target cells and by mean UMI among expressing cells. STEAP2 detection fell within the matched-gene distributions under both schemes: its detection percentiles were 0.13 and 0.39 in GSE131907 and 0.30 and 0.40 in GSE148071 (Supplementary Table S7). Thus, STEAP2 was not unusually sparse relative to genes with similar observed abundance, but the matched-gene benchmark cannot distinguish technical dropout from true on/off biological expression. These analyses shifted the single-cell conclusion toward iron-handling as the most reproducible STEAP2-associated program, with metabolic, EMT/YAP-like and inflammatory programs treated as context-dependent features.

### 3.5. CNV Filtering Preserves STEAP2-Associated Programs but Supports Marker Rather than Driver Wording

We used lightweight inferCNV-based sensitivity analyses to evaluate whether STEAP2-associated programs persisted after focusing on higher-confidence CNV-positive malignant or epithelial-like cells. In GSE131907, the STEAP2-associated signal remained detectable after CNV filtering, although effect sizes changed relative to the full malignant-cell analysis (Figure 2e; Supplementary Table S5). The metabolic signal remained visible after filtering, whereas EMT-like and CAF/ECM-like effects were weaker.

These CNV-aware results positioned STEAP2 as a marker embedded in a broader malignant epithelial copy-number context. They did not support treating STEAP2 as an upstream determinant of the observed state. Therefore, we next tested whether STEAP2 expression was simply a surrogate for 7q copy-number gain or retained non-redundant transcriptional information.

### 3.6. GSE148071 Supports Iron-Handling, IFN/MHC and EMT-like Features in CNV-High Epithelial-like Cells

We assessed the STEAP2-associated state in the independent GSE148071 NSCLC single-cell cohort. Because this dataset lacked author-provided malignant-cell labels comparable to GSE131907, epithelial-like cells were inferred from marker expression and filtered by lightweight inferCNV (Figure 3a, Figures S2 and S3; Supplementary Table S2). The resulting CNV-high epithelial-like compartment contained 6912 cells, including 820 STEAP2-positive and 6092 STEAP2-negative cells.

In this validation compartment, STEAP2-positive cells showed the strongest pooled effect for iron-handling genes (d = 1.067). EMT/YAP-like and IFN/MHC signatures were also higher in STEAP2-positive cells (EMT/YAP-like d = 0.452; IFN/MHC d = 0.296; Figure 3b; Supplementary Table S6). In contrast, proliferation and stemness-like scores were lower in STEAP2-positive cells (proliferation d = −0.244; stemness-like d = −0.325). These data shifted the validation emphasis toward iron-handling, inflammatory antigen-presentation, partial EMT-like features, and a lower-proliferative state.

Differential-expression and GSEA analyses supported the signature-level findings. STEAP2-positive CNV-high epithelial-like cells were enriched for interferon alpha response, interferon gamma response, EMT, TNF/NF-kB signaling, unfolded protein response and iron uptake/transport pathways (Figure 3c,d; Supplementary Table S6). E2F, G2M, MYC, oxidative phosphorylation, and reactive oxygen species-related programs were relatively enriched in STEAP2-negative cells.

Sample-level robustness analyses further supported the validation signal. Iron-handling was the most sample-robust feature in GSE148071, with consistent direction across leave-one-sample-out runs and paired-sample comparisons. EMT/YAP-like and IFN/MHC signals were stable in pooled and leave-one-sample-out analyses, but more heterogeneous at the paired-sample level (Figure 3e; Supplementary Table S7). Thus, GSE148071 supported key features of the STEAP2-associated state while showing a stronger iron-handling and inflammatory emphasis than GSE131907.

### 3.7. A Pre-Specified 7q Benchmark Supports STEAP2 as a Top-Decile Candidate Marker but Not the Strongest Regional Gene

**Figure 4:**
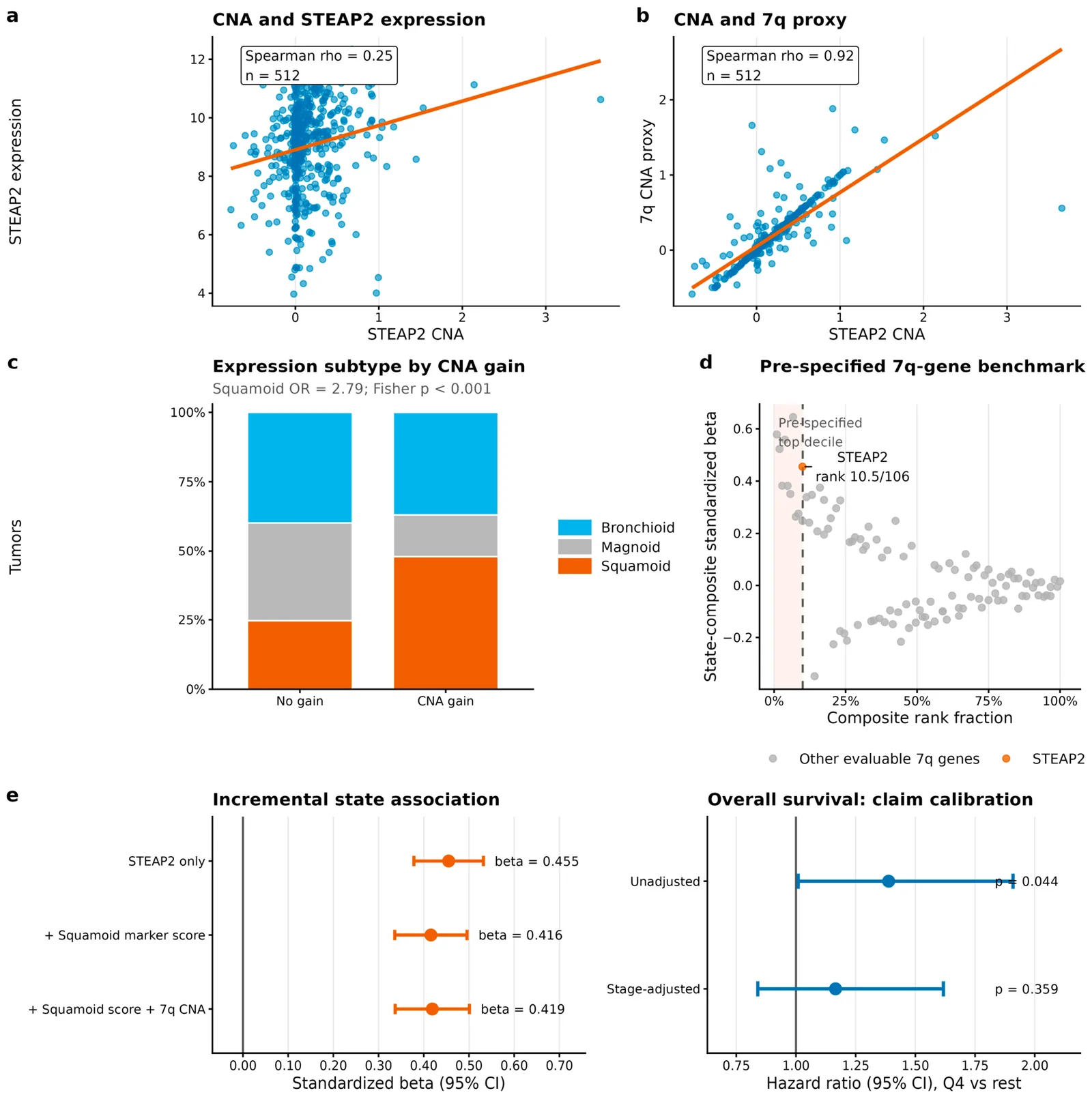
TCGA copy-number and 7q benchmark analyses support STEAP2 as a CNA-associated candidate state marker. (**a**,**b**) Associations between STEAP2 copy-number alteration (CNA), STEAP2 expression, and a 7q expression proxy in TCGA-LUAD. Correlations were evaluated using Spearman correlation. (**c**) TCGA-LUAD expression subtype distribution by STEAP2 CNA gain status. Bars show subtype composition in tumors with or without STEAP2 copy-number gain. (**d**) Pre-specified benchmark of all 106 evaluable genes in the STEAP2-centered 7q window for association with the state-composite score. The top-decile region is indicated, and STEAP2 is highlighted at rank 10.5 of 106 (rank fraction, 0.099). (**e**) Claim-calibration analyses. The state-association panel shows standardized STEAP2 coefficients and 95% confidence intervals before and after adjustment for the 7q CNA proxy and canonical Squamoid marker score. The survival panel shows unadjusted and stage-adjusted hazard ratios for STEAP2-high Q4 versus other TCGA-LUAD tumors. CNA, copy-number alteration; LUAD, lung adenocarcinoma.

Then, we examined TCGA-LUAD copy-number data to test the relationship between STEAP2 expression and 7q/CNA context. STEAP2 copy-number gain was present in 215 of 512 tumors (42.0%). STEAP2 copy-number correlated with STEAP2 expression (Spearman rho = 0.249, *p* < 0.001) and strongly correlated with a 7q expression proxy (rho = 0.919, *p* < 0.001) (Figure 4a,b; Supplementary Table S8). These results confirmed that STEAP2 expression is linked to a broader 7q/CNA background.

To test whether STEAP2 was an arbitrary 7q passenger gene, we benchmarked all evaluable genes in a pre-defined STEAP2-centered 7q window. Of 320 regional genes, 106 were evaluable in TCGA expression data. STEAP2 ranked 10.5 of 106 for association with a state-composite score, corresponding to a rank fraction of 0.099. Under the pre-specified decision rule, this placed STEAP2 in the top decile of evaluable 7q genes. This result supports STEAP2 as a top-decile candidate marker by rank threshold, but the margin was narrow and does not justify calling STEAP2 the strongest 7q marker.

STEAP2 copy-number gain was associated with Squamoid subtype enrichment (Figure 4c; Supplementary Table S8). However, the bulk Squamoid association was partly dependent on 7q/CNA context: STEAP2 remained associated with a continuous state-composite score after adjustment for the 7q CNA proxy and a canonical Squamoid marker score (standardized beta = 0.419, *p* = 1.33 × 10^−21^), whereas the association with binary Squamoid subtype became borderline after adding the 7q proxy. These results separate two claims. STEAP2 contains non-redundant state-composite information beyond 7q and canonical Squamoid markers, but its Squamoid subtype association should be interpreted as CNA-associated rather than fully independent.

### 3.8. Cross-Cohort Integration Identifies Shared Pathways and Context-Dependent Regulon Programs

**Figure 5:**
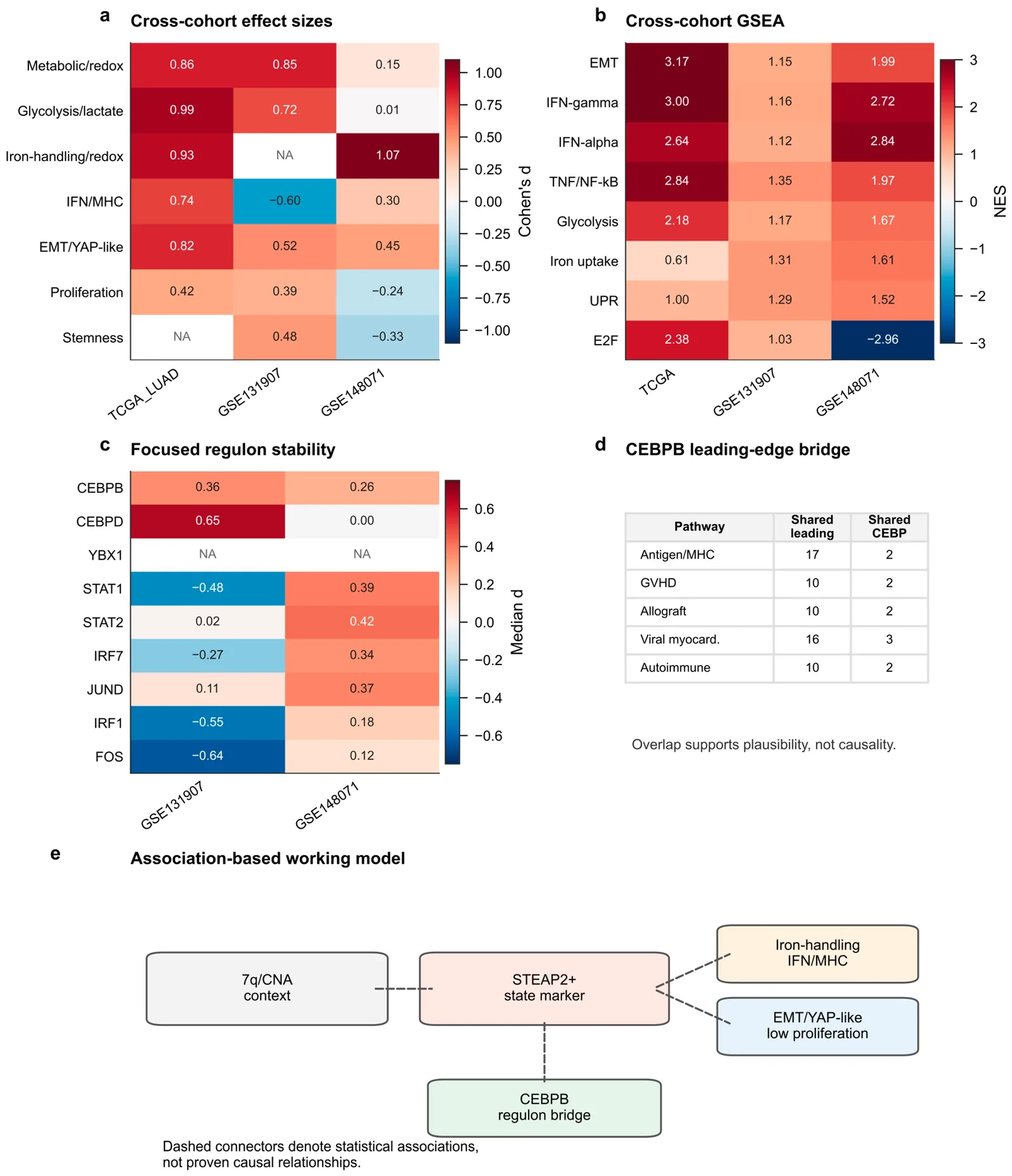
Cross-cohort integration and focused regulon analysis define shared and context-dependent STEAP2-associated programs. (**a**) Cross-cohort signature effect-size comparison across TCGA-LUAD, GSE131907, and GSE148071. Values summarize STEAP2-high or STEAP2-positive comparisons for metabolic/redox, glycolytic, iron-handling, IFN/MHC, EMT/YAP-like, proliferation, and stemness-like features where available. Because target-cell and reference-group definitions differ across datasets, effect sizes are interpreted within each contrast and not as directly interchangeable estimates. (**b**) Cross-cohort GSEA summary showing normalized enrichment scores for selected pathways across TCGA-LUAD, GSE131907, and GSE148071. Pathway-level patterns highlight shared interferon, antigen-presentation, iron-handling, and stress-response features. (**c**) Focused pySCENIC regulon bootstrap stability analysis in GSE131907 and GSE148071. Values summarize regulon effect sizes and positive-direction stability across bootstrap runs. (**d**) CEBPB and CEBP-family leading-edge overlap summary. Overlap was evaluated between inferred regulon targets and leading-edge genes from shared GSEA pathways. (**e**) Association-based working model of the STEAP2-associated LUAD/NSCLC malignant epithelial state. Dashed connectors denote statistical associations rather than proven causal relationships. pySCENIC, Python implementation of single-cell regulatory network inference and clustering.

We integrated TCGA-LUAD, GSE131907, and GSE148071 to separate shared from context-dependent STEAP2-associated features. Iron-handling was the most consistently reproduced single-cell feature, whereas metabolic/redox and EMT/YAP-like programs differed in strength across cohorts (Figure 5a; Supplementary Table S9). TCGA-LUAD and GSE131907 showed stronger metabolic and glycolytic associations. GSE148071 showed stronger iron-handling and IFN/MHC features. Stemness-like scoring was not consistently reproduced.

GSEA integration identified shared enrichment of antigen processing/presentation, interferon alpha/beta and interferon gamma signaling, iron uptake/transport, and endoplasmic-reticulum protein-processing pathways (Figure 5b and Figure S5; Supplementary Table S9). These pathways linked the TCGA bulk context, the GSE131907 metabolic/stress phenotype, and the GSE148071 inflammatory/iron-handling validation signal.

Focused pySCENIC regulon analysis with bootstrap stability assessment provided an upstream regulatory layer. In GSE131907, STEAP2-high malignant cells showed stable enrichment of CEBPD and CEBPB regulon activity, with YBX1 also elevated in the focused differential analysis (Figure 5c and Figure S4; Supplementary Table S10). In GSE148071, STEAP2-positive CNV-high epithelial-like cells showed stable enrichment of STAT1, STAT2, IRF7, JUND, and CEBPB regulon activity. CEBPB was therefore the most consistent cross-cohort regulon-level denominator. The broader regulon context differed between cohorts, with a more CEBP/stress-adaptive pattern in GSE131907 and a more STAT/IRF/AP-1 inflammatory pattern in GSE148071.

CEBP-family regulon targets partially overlapped with GSEA leading-edge genes from shared interferon, antigen-presentation, iron-handling, and stress-response pathways (Figure 5d; Supplementary Table S9). These inferred regulon results support a stress-associated regulatory correlate, not direct transcription-factor control. Together, the integrated analyses support an association-based model in which STEAP2 is a candidate marker of a 7q/CNA-associated iron-handling malignant epithelial state with context-dependent metabolic/stress-adaptive and inflammatory programs (Figure 5e).

As an exploratory supplementary analysis, focused transcript-based ligand-receptor scoring in GSE131907 nominated MIF-CD74/CXCR4, HLA/MHC and ICAM axes in STEAP2-high malignant cells (Supplementary Figure S6a–c; Supplementary Table S11). These scores indicate interaction potential only and do not establish spatial contact or functional signaling.

### 3.9. TCGA Survival Analyses Calibrate Clinical Interpretation

TCGA survival analyses then tested whether STEAP2 should be presented as a prognostic biomarker. STEAP2-high Q4 tumors showed a weak unadjusted overall-survival association compared with the remaining tumors (hazard ratio [HR] = 1.39, *p* = 0.044), but this was attenuated after stage adjustment (HR = 1.17, *p* = 0.359). Continuous STEAP2 expression was also not significant after stage adjustment. These results support state-identification wording rather than a prognostic-marker claim.

Together, these results define STEAP2 as a candidate marker of a 7q/CNA-associated malignant epithelial state linked to Squamoid subtype enrichment at the bulk-tumor level. The most reproducible single-cell feature is iron-handling, whereas metabolic, IFN/MHC, EMT/YAP-like and proliferation-related features are secondary or context-dependent.

## 4. Discussion

This study identifies STEAP2 as a candidate marker of a 7q/CNA-associated iron-handling malignant epithelial state in LUAD/NSCLC. Across TCGA-LUAD, GSE131907 and GSE148071, STEAP2-high or STEAP2-positive tumors and cells converged most consistently on iron-handling biology, while metabolic, IFN/MHC, EMT/YAP-like and proliferation-related features varied across cohorts and analytical levels. STEAP2-high tumors were enriched for the Squamoid expression subtype at the bulk-tumor level, a transcriptional LUAD subtype originally defined in independent expression cohorts [26]. The main advance is therefore not the rediscovery of 7q gain in lung cancer, but the calibrated definition of a single-gene candidate marker that captures part of a malignant-cell state within a broader CNA background.

This single-gene framing complements current single-cell atlases, where malignant epithelial states are usually defined by clustering, inferred copy-number profiles or multi-gene signatures [4,5,6]. Those definitions are informative but difficult to transfer to bulk cohorts or routine tissue assays. Experimental single-gene studies in LUAD, such as work linking PHLDA2 to ELK1-mediated progression, address causal function through perturbation [27]. Our analysis asks a narrower observational question: whether one transcript can recover a reproducible component of state structure across datasets. STEAP2 marked a minority malignant population in GSE131907, aligned with Squamoid subtype enrichment in TCGA-LUAD, and retained detectable state-composite information after CNA and canonical Squamoid marker adjustment.

The copy-number analyses refine this interpretation. STEAP2 expression was associated with STEAP2 copy-number gain and a broader 7q proxy, consistent with the importance of recurrent somatic copy-number alterations in LUAD [2,11]. However, copy-number effects on RNA and protein abundance can be modified by dosage compensation and other regulatory processes [12]. In a pre-specified 7q benchmark, STEAP2 ranked among the top decile of evaluable genes for association with the state-composite score, but only narrowly. STEAP2 expression also retained state-composite association after adjustment for the 7q CNA proxy and canonical Squamoid marker score. These results support STEAP2 as a non-redundant candidate state marker within 7q/CNA context, not as the strongest regional gene or a copy-number-independent driver.

Iron handling provides the strongest biological anchor for this state. STEAP2 has experimentally demonstrated ferrireductase and cupric-reductase activity [8], and STEAP-family metalloreduction contributes to cellular metal uptake and redox homeostasis [28]. In the revised sample-level analyses, iron-handling was directionally consistent in both single-cell cohorts, remained evident after removing STEAP2 from the score, and persisted after UMI/nGene adjustment. This pattern supports an iron-handling malignant epithelial program rather than a single-gene scoring artifact. It still leaves open to interpretation whether STEAP2 enzymatic activity directly contributes to this LUAD state; prior prostate cancer perturbation studies support a possible invasive role for STEAP2 [10], but LUAD-specific functional validation remains necessary.

The differences between GSE131907 and GSE148071 appear biologically and technically plausible rather than simply inconsistent. GSE131907 spans primary tumors, metastatic sites and pleural effusions, whereas GSE148071 comprises diagnostic samples from advanced NSCLC [5,6]. The former emphasized metabolic and glycolytic features; the latter emphasized iron-handling, IFN/MHC and EMT-like enrichment. This divergence may reflect cohort composition, sampling site, treatment context, tissue processing, annotation strategy or genuine context dependence, consistent with prior single-cell work showing lung cancer programs that vary across disease stage and treatment exposure [3,7].

Focused pySCENIC analysis with bootstrap stability assessment identified CEBPB as the most consistent cross-cohort regulon-level denominator. GSE131907 showed a more CEBP/stress-adaptive pattern, whereas GSE148071 showed stronger STAT1, STAT2, IRF7 and JUND enrichment. CEBPB expression can be induced by endoplasmic-reticulum stress and coupled to interferon-STAT signaling, and CEBPD can connect PERK-mediated stress responses to immunomodulatory chemokine expression in other contexts [29,30,31]. These observations position CEBPB as a stress-associated regulon correlate across cohorts. They do not establish CEBPB as a mechanistic bridge or prove transcription-factor control in STEAP2-positive LUAD cells.

The EMT/YAP-like and immune-interaction findings also require calibration. EMT in cancer is a spectrum of intermediate states rather than a binary switch [32], and LUAD EMT-related transcription can overlap with inflammatory and immune-checkpoint programs [33]. Experimental work on LGR4/NF-kB illustrates how an inflammatory progression mechanism can be established through functional validation [34]; in contrast, NF-kB enrichment in our study is associative. YAP can influence lineage plasticity and adeno-to-squamous transitions in experimental models [35], but canonical YAP/TEAD target evidence was incomplete in our single-cell results; we therefore use EMT/YAP-like as a transcriptional label, not as proof of YAP/TEAD activation. Similarly, focused CellPhoneDB-style scoring nominated MIF-CD74/CXCR4, HLA/MHC and ICAM axes, which are biologically plausible in NSCLC [36,37,38,39], but transcript-based ligand-receptor scores do not establish spatial contact or functional signaling.

The broader clinical implications of STEAP2 should be separated into diagnostic, prognostic and therapeutic domains. Diagnostically, STEAP2 could be evaluated as a tissue-compatible indicator of the 7q/CNA-associated iron-handling state, but this study did not test diagnostic sensitivity, specificity or discrimination from a non-malignant epithelium and therefore does not establish a diagnostic biomarker. Prognostically, TCGA survival associations were attenuated after stage adjustment, arguing against independent prognostic value. Therapeutically, the iron-handling association nominates a testable hypothesis that STEAP2-positive cells may have distinct metal/redox dependencies, but no perturbation or drug-response experiment demonstrates that STEAP2 is a functional vulnerability or treatment target. Human Protein Atlas resources support antibody-based detection in lung cancer tissue [40,41,42] (Supplementary Table S12), but available IHC data do not resolve the single-cell-inferred minority malignant-cell pattern. Thus, the current translational value is state identification and hypothesis generation rather than clinical diagnosis, prognosis or therapeutic selection.

Several limitations define the boundary of the study. The analysis used retrospective public datasets with different platforms, sampling contexts and annotation depth. GSE148071 lacked author-defined malignant-cell labels comparable to GSE131907 and should be interpreted as validation in CNV-high epithelial-like cells. InferCNV-based filtering was a lightweight sensitivity framework rather than definitive genomic copy-number profiling. Focused pySCENIC and ligand-receptor analyses generated regulatory and communication hypotheses from transcript abundance and do not replace perturbation, spatial or functional validation. Spatial reconstruction methods can infer spatial organization from scRNA-seq under explicit modeling assumptions [43], but no spatial inference or spatially resolved validation was performed here. HPA immunohistochemistry supports detectability, but not spatial organization or minority-cell-state validation.

## 5. Conclusions

STEAP2 is a candidate marker of a 7q/CNA-associated iron-handling malignant epithelial state in LUAD/NSCLC. The state is associated with Squamoid enrichment at the bulk-tumor level and has context-dependent metabolic, IFN/MHC, EMT/YAP-like and proliferation-related features. STEAP2 may make one component of this complex state more accessible to bulk and tissue-based studies, but prospective spatial, protein-level and functional validation is required before clinical use.

## Figures and Tables

**Table 1:** Public cohorts and analysis design. This table summarizes cohort roles, sample or cell numbers, STEAP2 grouping strategies, copy-number or malignancy definitions, and claim limitations.

Cohort_or_Resource	Key_Analytical_Role	Data_Type	Sample_or_Cell_Number	Target_Population	Steap2_Grouping	Cnv_or_Malignancy_Definition	Main_Analysis_Performed	Claim_Limitation
TCGA-LUAD	Bulk context, 7q benchmark, incremental models, and survival claim calibration	Bulk RNA-seq, expression subtype, GISTIC2 copy-number, and clinical survival data	512–517 tumors for expression/CNA models; 496 tumors in the stage-adjusted Q4-versus-rest survival model	TCGA-LUAD primary tumor samples	STEAP2-low Q1, middle Q2–Q3, STEAP2-high Q4; continuous standardized expression in sensitivity models	Matched GISTIC2 gene-level CNA; pre-defined STEAP2-centered 7q window and 7q CNA proxy	Squamoid enrichment, module scoring, pre-ranked GSEA, 7q all-gene benchmark, Squamoid-marker/CNA incremental models, and Cox survival sensitivity analyses	Bulk-tumor association only; STEAP2 was not independently prognostic after stage adjustment and the Squamoid association was partly 7q/CNA-dependent
GSE131907	Single-cell discovery with author-annotated malignant cells and sample-level robustness	Single-cell RNA-seq	58 samples in the source cohort; 24,784 author-annotated malignant cells; 19 samples eligible for the paired iron-handling analysis	Author-annotated malignant cells	Primary harmonized analysis: STEAP2-positive versus negative; q50, q75, q90 and continuous expression as sensitivity definitions	Author malignant-cell annotation; lightweight inferCNV used only for CNV sensitivity filtering	DEG, signature scoring, pre-ranked GSEA, within-sample paired analysis, UMI/nGene-adjusted mixed-effects models, cutoff/continuous sensitivity, and focused supplementary ligand-receptor scoring	Associative discovery; detection fraction is depth-dependent; lightweight inferCNV and transcript-based communication scores do not establish genomic CNA, spatial contact, or causality
GSE148071	Independent single-cell validation in a conservatively defined CNV-high epithelial-like compartment	Single-cell RNA-seq	42 samples in the source cohort; 6912 CNV-high epithelial-like cells (820 STEAP2-positive and 6092 negative); 24 samples eligible for the paired iron-handling analysis	Marker-inferred CNV-high epithelial-like cells	Primary harmonized analysis: STEAP2-positive versus negative; q50, q75, q90 and continuous expression as sensitivity definitions	Marker-inferred epithelial-like cells and lightweight inferCNV CNV-high filter	Full DEG and GSEA, signature effects, within-sample paired analysis, UMI/nGene-adjusted mixed-effects models, cutoff/continuous sensitivity, and focused pySCENIC	Validation population is not author-annotated malignant; iron-handling replicated, whereas metabolic, IFN/MHC, EMT/YAP-like, and proliferation-related programs were context-dependent

## Data Availability

The datasets analyzed in this study are publicly available from TCGA, GEO and the Human Protein Atlas. The single-cell RNA-seq cohorts were GSE131907 and GSE148071; GSE126044 was retained only as preliminary supplementary context. Processed tables supporting the figures are provided in the supplementary tables and source-data files prepared with this revision. Analysis scripts and processed outputs are organized with analysis-specific source-data tables. A public code release will exclude large public raw-data files and provide download instructions for the corresponding repositories.

## Supplementary Materials

The following supporting information can be downloaded at: https://www.mdpi.com/article/10.3390/cancers18183050/s1, Figure S1: Quality-control analyses for the GSE131907 discovery contrast; Figure S2: Single-cell cohort composition and STEAP2-positive target-cell fractions; Figure S3: Epithelial-like cell definition and lightweight inferCNV quality control in GSE148071; Figure S4: Focused pySCENIC regulon differential activity and bootstrap stability; Figure S5: Full pre-ranked GSEA context across cohorts and gene-set libraries; Figure S6: Focused ligand-receptor and IHC analyses provide exploratory context; Figure S7: Signature scoring note and gene-set definitions; Table S1: Signature score definitions and gene sets; Table S2: Dataset inclusion, target-cell definitions and key cutoffs; Table S3: TCGA-LUAD STEAP2 strata, expression subtype, module scores and GSEA; Table S4: GSE131907 malignant-cell differential expression and signature effects; Table S5: GSE131907 quality-control and CNV sensitivity analyses; Table S6: GSE148071 CNV-high epithelial-like validation; Table S7: Single-cell sample-level, cutoff and depth-adjusted robustness analyses; Table S8: TCGA copy-number, 7q benchmark, Squamoid-marker comparison and survival analyses; Table S9: Cross-cohort signature and pathway integration; Table S10: Focused pySCENIC regulon analysis; Table S11: Focused ligand-receptor analysis in GSE131907; Table S12: HPA/IHC feasibility and supplementary exploratory ICI context; Table S13: Figure source-data manifest.
