# Supplementary material for: STEAP2 Is a Candidate Marker of a 7q/CNA-Associated Iron-Handling Malignant Epithelial State in Lung Adenocarcinoma
Source: Cancers (Basel). 2026 Sep 20;18(18):3050. doi: 10.3390/cancers18183050 (PMC13604872; doi:10.3390/cancers18183050)
Supplement: Supplementary file 1 [file cancers-18-03050-s001.zip › Supplementary_Methods.pdf]

Supplementary Methods

Preliminary exploratory immunotherapy-response analysis

GSE126044 was obtained from GEO and analyzed only as a small exploratory anti-PD-1-treated NSCLC RNA-seq cohort [1,2,3]. Raw counts were converted to log2 counts per million, genes were z-scored across samples, and module scores were calculated as the mean z-scored expression of available signature genes. Response-associated differences were summarized using group means, Cohen's d and exact two-sided permutation P values. A fresh-sample-only sensitivity analysis was performed because the cohort included mixed sample materials. The small sample size and material imbalance precluded interpretation as a response-biomarker model, and these results were not used to support main-text clinical claims.

References

1. Edgar R, Domrachev M, Lash AE. Gene Expression Omnibus: NCBI gene expression and hybridization array data repository. Nucleic Acids Research. 2002;30(1):207-210. PMID:11752295.

2. Barrett T, Wilhite SE, Ledoux P, et al. NCBI GEO: archive for functional genomics data sets--update. Nucleic Acids Research. 2013;41(Database issue):D991-D995. doi:10.1093/nar/gks1193. PMID:23193258.

3. Cho JW, Hong MH, Ha SJ, et al. Genome-wide identification of differentially methylated promoters and enhancers associated with response to anti-PD-1 therapy in non-small cell lung cancer. Experimental & Molecular Medicine. 2020;52(9):1550-1563. doi:10.1038/s12276-020-00493-8. PMID:32879421.
